# Distinct facial expressions represent pain and pleasure across cultures

**DOI:** 10.1073/pnas.1807862115

**Published:** 2018-10-08

**Authors:** Chaona Chen, Carlos Crivelli, Oliver G. B. Garrod, Philippe G. Schyns, José-Miguel Fernández-Dols, Rachael E. Jack

**Affiliations:** ^a^School of Psychology, College of Science and Engineering, University of Glasgow, Glasgow G12 8QB Scotland, United Kingdom;; ^b^Institute of Neuroscience and Psychology, College of Medical, Veterinary and Life Sciences, University of Glasgow, Glasgow G12 8QB Scotland, United Kingdom;; ^c^Institute for Psychological Science, School of Applied Social Sciences, De Montfort University, Leicester LE1 9BH, United Kingdom;; ^d^Departamento de Psicología Social y Metodología, Facultad de Psicología, Universidad Autónoma de Madrid, 28049 Madrid, Spain

**Keywords:** pain, orgasm, facial expressions, culture, data-driven methods

## Abstract

Real-world studies show that the facial expressions produced during pain and orgasm—two different and intense affective experiences—are virtually indistinguishable. However, this finding is counterintuitive, because facial expressions are widely considered to be a powerful tool for social interaction. Consequently, debate continues as to whether the facial expressions of these extreme positive and negative affective states serve a communicative function. Here, we address this debate from a novel angle by modeling the mental representations of dynamic facial expressions of pain and orgasm in 40 observers in each of two cultures (Western, East Asian) using a data-driven method. Using a complementary approach of machine learning, an information-theoretic analysis, and a human perceptual discrimination task, we show that mental representations of pain and orgasm are physically and perceptually distinct in each culture. Cross-cultural comparisons also revealed that pain is represented by similar face movements across cultures, whereas orgasm showed distinct cultural accents. Together, our data show that mental representations of the facial expressions of pain and orgasm are distinct, which questions their nondiagnosticity and instead suggests they could be used for communicative purposes. Our results also highlight the potential role of cultural and perceptual factors in shaping the mental representation of these facial expressions. We discuss new research directions to further explore their relationship to the production of facial expressions.

## Significance

Humans often use facial expressions to communicate social messages. However, observational studies report that people experiencing pain or orgasm produce facial expressions that are indistinguishable, which questions their role as an effective tool for communication. Here, we investigate this counterintuitive finding using a new data-driven approach to model the mental representations of facial expressions of pain and orgasm in individuals from two different cultures. Using complementary analyses, we show that representations of pain and orgasm are distinct in each culture. We also show that pain is represented with similar face movements across cultures, whereas orgasm shows differences. Our findings therefore inform understanding of the possible communicative role of facial expressions of pain and orgasm, and how culture could shape their representation.

Studies of real-world scenarios show that people experiencing intense negative or positive affect—for example, pain or orgasm—spontaneously produce facial expressions that are very similar (1–4). This finding is counterintuitive, because facial expressions are widely considered to be a powerful tool for human social communication and interaction, including the socially relevant states of extreme positive and negative affect (5–7). Consequently, the extent to which such intense states can be accurately inferred from facial expressions remains a central debate in the cognitive sciences that involves input from psychological, ethological, pragmatic, and information-theoretic approaches (1, 3, 8–13).

Here, we address this debate from a novel angle. Using a data-driven reverse-correlation approach, we model the dynamic mental representations of facial expressions of intense positive and negative affect—physical pain and sexual pleasure—in individuals from two cultures. We take this approach for two reasons. First, mental representations are built from encounters with the external environment, either directly or vicariously (e.g., learning cultural concepts), and are thus used to predict and interpret the environment (14). Understanding the content of these representations can therefore inform what an individual might have learned from their real-world interactions. Second, data-driven approaches enable a broader range of facial expressions to be tested as representative of these intense affects because they are sampled agnostically from a less constrained array than traditional theory-driven approaches (15) and without the inevitable complexities of accurately measuring facial expressions in the wild (e.g., see ref. 16).

To examine whether mental representations of facial expressions of physical pain and sexual pleasure (i.e., orgasm) are distinguishable or not, we modeled these representations in individuals from two cultures (Western and East Asian; *Methods*, *Observers*). For brevity, we now refer to these mental representations as “facial expression models of pain and orgasm.” We then analyzed how distinguishable these facial expression models are within and across cultures using a complementary approach of machine learning, a human perceptual discrimination task, and an information-theoretic analysis. We also compared the facial expression models across cultures to identify any cross-cultural similarities and differences in the face movements that represent these extreme affective states.

**Fig. 1.**
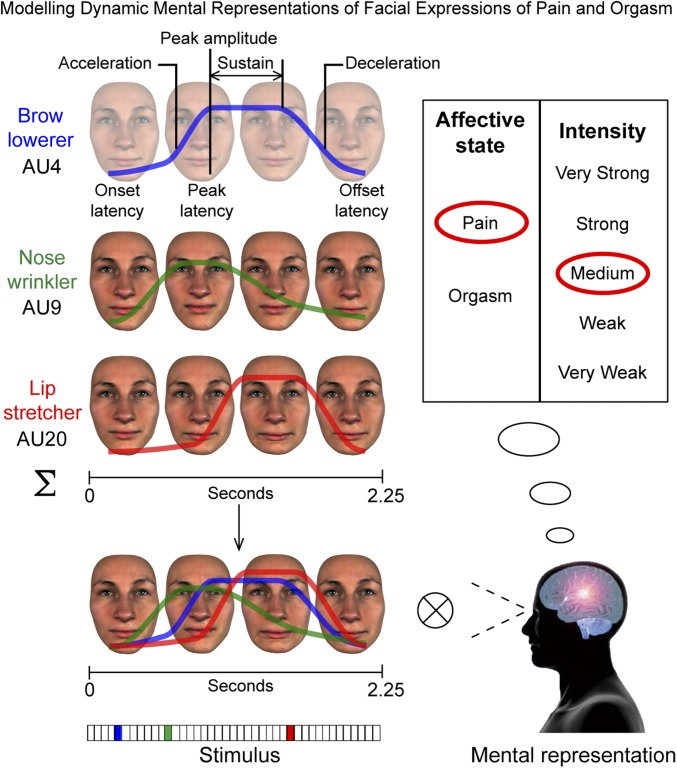
Modeling dynamic mental representations of facial expressions of pain and orgasm. Stimulus: On each experimental trial, a dynamic face movement generator (18) randomly selected a biologically feasible combination of individual facial movements called action units [AUs (19)] from a core set of 42 AUs (here, brow lowerer, AU4, color-coded in blue; nose wrinkler, AU9, in green; and lip stretcher, AU20, in red). A random movement is then assigned to each AU individually by selecting random values for each of seven temporal parameters (i.e., onset latency, acceleration, peak amplitude, peak latency, sustainment, deceleration, and offset latency; see labels illustrating the blue curve). The randomly activated AUs are then combined and displayed on a photorealistic face identity to produce a random facial animation, shown here by the sequence of four face images. The color-coded vector below shows the three AUs randomly selected on this example trial. Mental representation: The observer viewed the facial animation and, if the dynamic face movements correlated with their mental representation (i.e., prior knowledge) of a facial expression of pain or orgasm, they categorized it accordingly (here, pain) and rated its intensity on a five-point scale from very weak to very strong (here, medium). Otherwise, the observer selected other. Each observer (40 per culture—Western and East Asian—heterosexual) categorized 3,600 such facial animations, each displayed on same-race, sex-opposite faces and presented in random order across the experiment.

To derive these facial expression models, we used a data-driven technique based on reverse correlation (17) that generates face movements agnostically—that is, with minimal assumptions about which face movements represent which messages to whom (15, 18). Fig. 1 illustrates this procedure using an example trial. On each trial, a dynamic face movement generator (18) randomly selected a combination of individual face movements called action units [AUs (19)] from a core set of 42 AUs (minimum 1, maximum 4, and median 3 AUs selected on each trial). In the example trial of Fig. 1, three AUs are randomly selected: brow lowerer (AU4) color-coded in blue, nose wrinkler (AU9) color-coded in green, and lip stretcher (AU20) color-coded in red. A random movement is then assigned to each AU separately using seven randomly selected values, one for each temporal parameter of onset latency, acceleration, peak amplitude, peak latency, sustainment, deceleration, and offset latency (see labels illustrating the blue curve). The randomly activated AUs are then combined and displayed on a photorealistic face identity to produce a random facial animation (duration 2.25 s). An example is shown in Fig. 1 using a sequence of four images (Movie S1 shows the facial animation generation procedure represented in Fig. 1). Observers in each culture viewed the resulting facial animation, and if the face movements matched their mental representation of a facial expression of “pain” or “orgasm,” they categorized it accordingly (here, pain) and rated its intensity on a five-point scale from “very weak” to “very strong” (here, “medium”). Otherwise, if the facial animation did not match the observer’s mental representation of pain or of orgasm, they selected “other.” Each observer completed 3,600 such trials, resulting in a set of facial animations for pain and for orgasm. We can then build a statistical relationship between the face movements on each trial and the observer’s responses. This analysis thus produces a model of the face movements that represent pain and orgasm in the mind of each observer (see *SI Appendix*, *Modeling Dynamic Mental Representations of Facial Expressions of Pain and Orgasm* for full details; see Movie S2 for an illustration).

We used this technique to model the dynamic mental representations of facial expressions of pain and orgasm in each of 40 observers per culture (see Movie S3 for examples of these models in each culture). To objectively examine the distinctiveness of these facial expression models, we used machine learning (a Bayesian classifier) and an information-theoretic analysis using the measurement of mutual information. We also asked new sets of cultural observers to discriminate each facial expression model in a perceptual discrimination task (see *Methods*, *Physical and Perceptual Distinctiveness of the Facial Expression Models of Pain and Orgasm* for full details). Our complementary analyses show that, in each culture, the facial expression models of pain and orgasm are both physically and perceptually distinct. Cross-cultural comparisons also show differences in the facial expression models of orgasm, including wide-open eyes among Westerners and smiling in East Asians. In contrast, facial expression models of pain are similar across cultures. We discuss the implications of our data-driven findings of distinct mental representations of the facial expressions of pain and orgasm with respect to the similarity of their production.

## Results

**Fig. 2.**
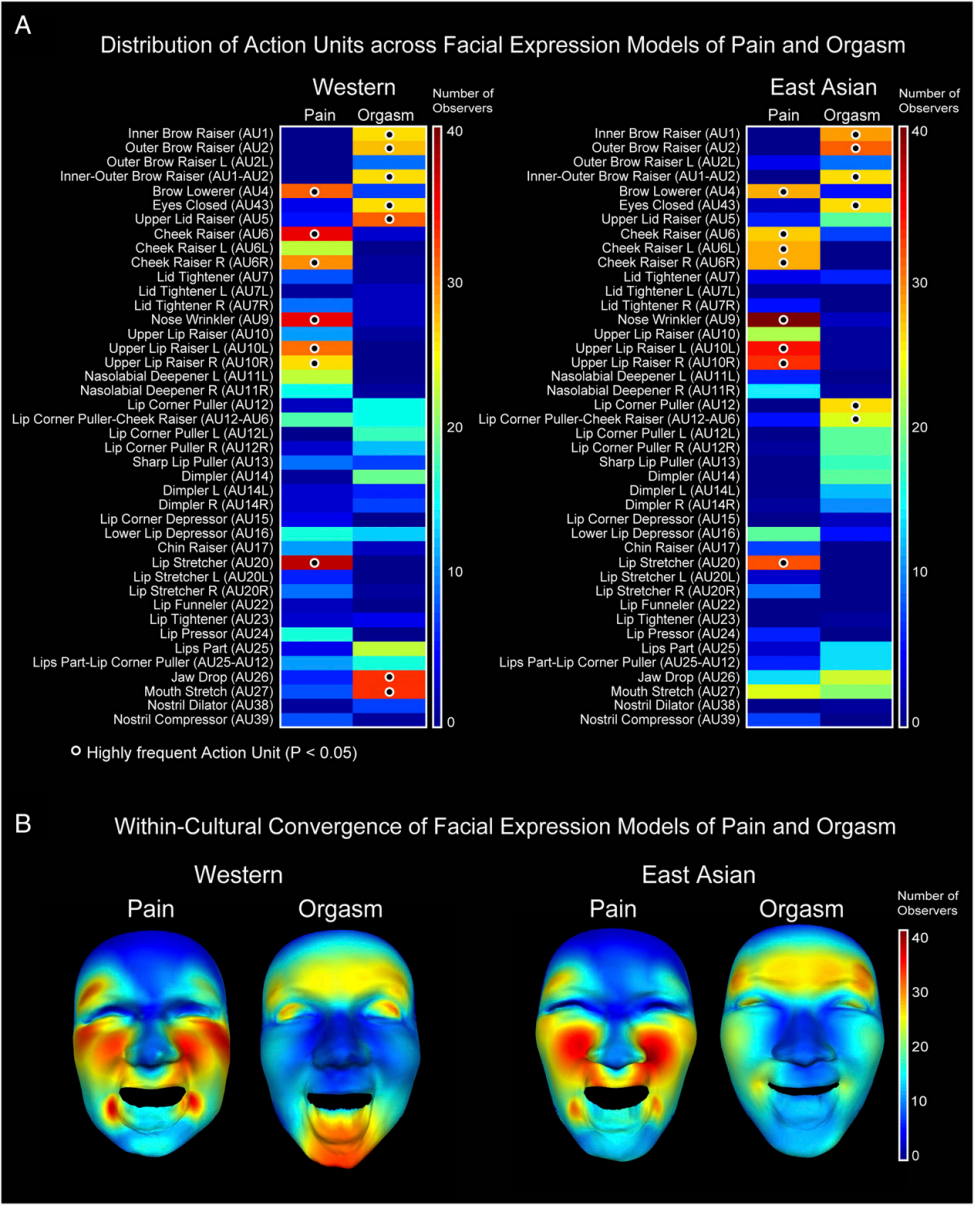
Distribution of action units across facial expression models of pain and orgasm and their convergence across observers within each culture. (*A*) Distribution of AUs across facial expression models of pain and orgasm. For each culture—Western (*Left*) and East Asian (*Right*)—and pain and orgasm separately, the color-coded matrix shows the number of individual observer facial expression models (maximum 40) with each AU (see labels on the left). Warmer colors indicate more observers; cooler colors indicate fewer observers (see color bar on the right). A black dot indicates the AUs that are highly frequent (one-tailed, *P* < 0.05) across all individual observer models as determined using a Monte Carlo simulation method (*SI Appendix*, *Highly Frequent Action Units*). (*B*) Within-culture convergence of facial expression models of pain and orgasm. Color-coded face maps show for each culture and affective state the number of individual observer facial expression models with each AU using the same color coding as in *A*.

Using the above data-driven method, we modeled a total of 160 dynamic mental representations of facial expression of pain and orgasm (40 observers × 2 cultures × 2 affective states). We henceforth refer to these as “models.” Each model is represented as a 1 × 42-dimensional binary vector detailing the AUs that are significantly associated with the perception of each affective state (i.e., pain or orgasm) plus seven values detailing the temporal dynamics of each significant AU (see *Methods*, *Modeling Dynamic Mental Representations of Facial Expressions of Pain and Orgasm* for full details). Fig. 2 shows the distribution of significant AUs across all 40 individual observer models for pain and orgasm in each culture separately. Each distribution is represented as a color-coded matrix (Fig. 2*A*) with corresponding face maps (Fig. 2*B*). We also compared the facial expression models with the AUs reported in studies of the production of facial expressions during physical pain (20–30) or orgasm (1–3), which showed that they are generally very similar (see *SI Appendix*, *Comparison of the Mental Representations and Productions of Facial Expressions of Pain and Orgasm* and Fig. S1 for full details). We then identified the AUs that appear most frequently across the 40 models of pain and orgasm in each culture separately using a Monte Carlo simulation method (see *SI Appendix*, *Highly Frequent Action Units* for full details). Fig. 2*A* indicates these highly frequent AUs with a black dot. A casual inspection of Fig. 2 suggests that, in each culture, the facial expression models of pain and orgasm are distinct. To objectively test the distinctiveness of the facial expression models in each culture, we built and tested a Bayesian model of the classification task. We also tested whether humans could perceptually discriminate these facial expression models by asking a new set of observers in each culture to perform a perceptual discrimination task.

### Bayesian Classification of Facial Expression Models of Pain and Orgasm in Each Culture.

**Fig. 3.**
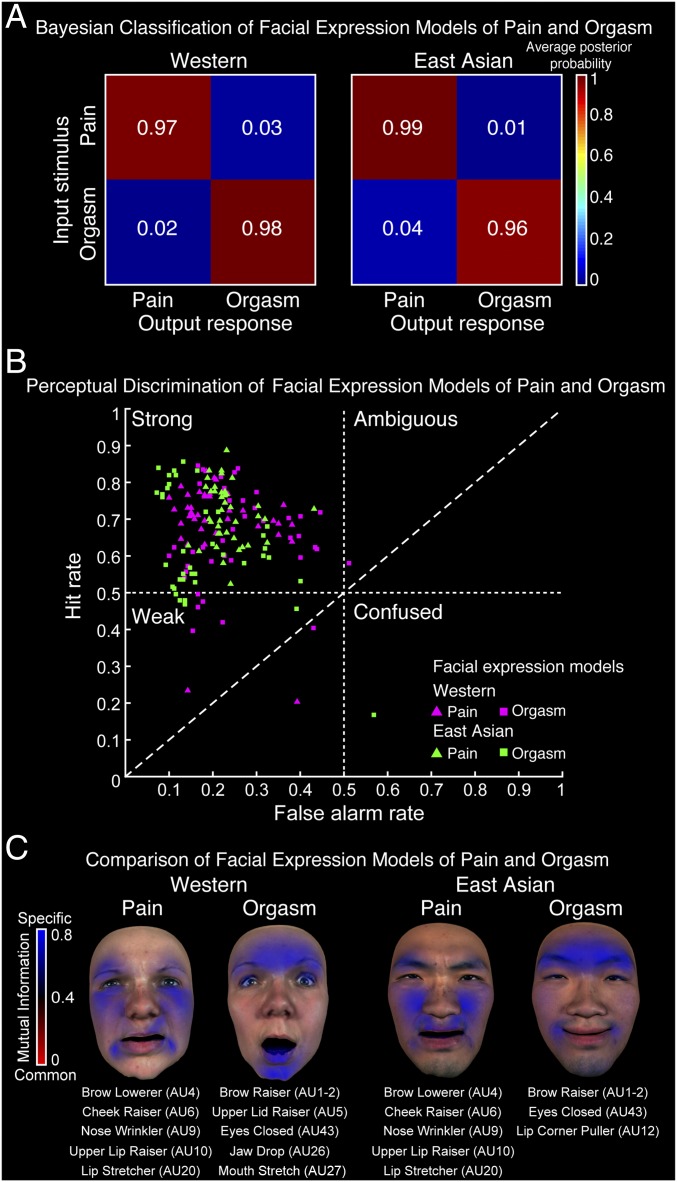
Distinctiveness of the facial expression models of pain and orgasm in each culture. (*A*) Bayesian classification of facial expression models of pain and orgasm. Color-coded matrices show for each culture the average posterior probability that a facial expression model of pain or orgasm (input stimulus) is classified as pain or orgasm (output response). Red shows high posterior probability of classification; blue indicates low posterior probability (see color bar to the right). Exact values are shown in each square. Diagonal squares show correct classifications; off-diagonal squares show incorrect classifications. The red-colored diagonal squares show that in each culture the facial expression models of pain and orgasm are discriminated with high accuracy. (*B*) Perceptual discrimination of facial expression models of pain and orgasm. Each color-coded shape represents an individual observer facial expression model (*n* = 40 per culture) plotted according to its hit rate and its false alarm rate (i.e., *d*-prime value, which is a measure of perceptual discrimination). Magenta points represent Western models, and green represents East Asian models; triangles represent pain, and squares represent orgasm (see legend in the bottom right). The four quadrants (delimited by dashed lines) indicate the different response types based on hit and false alarm rates (see labels in each quadrant). The diagonal dashed line represents an equal rate of hits and false alarms. The distribution of the data points in the upper left-hand quadrant shows that the vast majority of facial expression models from both cultures are discriminated well with virtually no instances of ambiguity or confusion. (*C*) Comparison of facial expression models of pain and orgasm. To objectively identify which face movements (i.e., AUs) are specific to or common across pain and orgasm in each culture, we used mutual information (*Methods*, *Comparison of the Facial Expression Models of Pain and Orgasm*). For each culture, blue coloring on the face maps shows the AUs that are specific to pain or to orgasm (significantly high MI, *P* < 0.05), and red coloring shows those that are common across pain and orgasm (low MI; see color bar to the left). Homogeneous blue coloring shows that in each culture the facial expression models of pain and orgasm have no commonalities; AUs specific to pain and to orgasm in each culture are listed below each face map.

To objectively test the distinctiveness of the facial expression models of pain and orgasm in each culture, we built a Bayesian model of the discrimination task using a split-half method (*SI Appendix*, *Bayesian Classification of Facial Expression Models*). This analysis computes the average posterior probability that a pain or orgasm facial expression model (input stimulus) is classified as either pain or orgasm (output response). Fig. 3*A* shows the results for each culture, where dark red shows high posterior probability of classification for pain and orgasm, and blue indicates low posterior probability (see color bar to the right). Exact values are shown in each square. The diagonal squares show correct classifications; off-diagonal squares show incorrect classifications. As shown in Fig. 3*A* by the red-colored diagonal squares in each culture, the Bayesian model consistently discriminated the facial expression models of pain and orgasm with very little confusion (average classification performance: Western: pain *M* = 0.97, *SD* = 0.004; orgasm *M* = 0.98, *SD* = 0.002; East Asian: pain *M* = 0.99, *SD* = 0.001; orgasm *M* = 0.96, *SD* = 0.005). These results show that in each culture, mental representations of the facial expressions of pain and orgasm are consistently different.

### Perceptual Discrimination of the Facial Expression Models of Pain and Orgasm in Each Culture.

Having demonstrated that mental representations of facial expressions of pain and orgasm are objectively distinct in each culture, we now ask whether they are perceptually discriminable—that is, they convey pain and orgasm to other cultural observers. To test this, we asked a new set of observers from each culture to discriminate each facial expression model in a perceptual discrimination task (see *Methods*, *Physical and Perceptual Distinctiveness of the Facial Expression Models of Pain and Orgasm* for full details). Following the experiment, we computed the *d*-prime value (31, 32)—a measure of perceptual discrimination—of each individual facial expression model in each culture. Specifically, *d*-prime shows the extent to which a specific signal can be accurately distinguished from others by considering both the hit rates (i.e., accurately reporting the presence of a signal when it is present) and the false alarm rates (i.e., erroneously reporting the presence of a signal when another is present). This approach therefore safeguards against artificially high accuracy rates that can occur based on random responding (33–35). Fig. 3*B* shows the results where color-coded shapes represent individual observer facial expression models plotted according to their hit rate (*y* axis) and false alarm rate (*x* axis). Magenta points represent Western models, and green points represent East Asian models; triangles represent pain, and squares represent orgasm (see legend on bottom right). Dashed lines outline the four response types based on high/low hits and false alarm rates (see labels in each quadrant). For example, a high hit and low false alarm rate (top left-hand quadrant) indicates strong perceptual discrimination of the facial expression model (e.g., many “yes” responses when correctly matched, and few yes responses when incorrectly matched). In contrast, a high hit rate and a high false alarm rate (top right-hand quadrant) indicates that the facial expression model is ambiguous (e.g., many yes responses when the facial expression model appears with either pain or orgasm). The diagonal dashed line indicates equal hit and false alarm rates. As shown by the distribution of the data points primarily in the top left-hand quadrant, the majority of the facial expression models of pain and orgasm in each culture were discriminated well by human observers with virtually no instances of ambiguity or confusion. *SI Appendix*, Fig. S2 shows all individual observer pain and orgasm facial expression models displayed as face maps and ranked by *d*-prime for each culture separately.

### Revealing the Distinctive Face Movements in the Mental Representations of Pain and Orgasm.

The Bayesian classifier demonstrated the objective distinctiveness of the facial expression models of pain and orgasm in each culture. The perceptual discrimination task further demonstrated that observers in each culture could use these differences in the face movements to distinguish pain and orgasm. To precisely identify which face movements (i.e., AUs) are distinct to the facial expression models of pain and orgasm (or are common to them), we measured the relationship of each AU to pain and to orgasm using an information-theoretic analysis based on mutual information (MI; see *Methods*, *Comparison of the Facial Expression >Models of Pain and Orgasm* for full details). Specifically, MI measures the strength of the relationship between an AU (e.g., brow lowerer) and category labels (here, pain and orgasm). High MI values indicate a strong relationship (i.e., strong effect size)—that is, the AU is more commonly associated with pain than with orgasm, or vice versa. Low MI indicates a weak relationship—that is, the AU is associated with both pain and orgasm with similar frequency. We applied this analysis only to the highly frequent AUs (*SI Appendix*, *Highly Frequent Action Units*) to ensure that the specific and common face movements identified are representative of the set of 40 models of pain and orgasm in each culture. Therefore, AUs with high MI values and that are highly frequent in either pain or orgasm are considered to be specific AUs. Common AUs have low MI values and are highly frequent in both pain and orgasm. We established statistical significance of the MI values using a Monte Carlo method (see *Methods*, *Comparison of the Facial Expression Models of Pain and Orgasm* for full details). Fig. 3*C* shows the results. For each culture, color-coded face maps show the AUs that are specific to pain and to orgasm (blue, significantly high MI, *P* < 0.05) and those that are common (red, low MI; see color bar to the left). As shown by the homogeneous blue coloring, the facial expression models of pain and orgasm have no common AUs. The AUs specific to pain and to orgasm in each culture are listed below each face.

### Cross-Cultural Comparison of the Facial Expression Models of Pain and Orgasm.

**Fig. 4.**
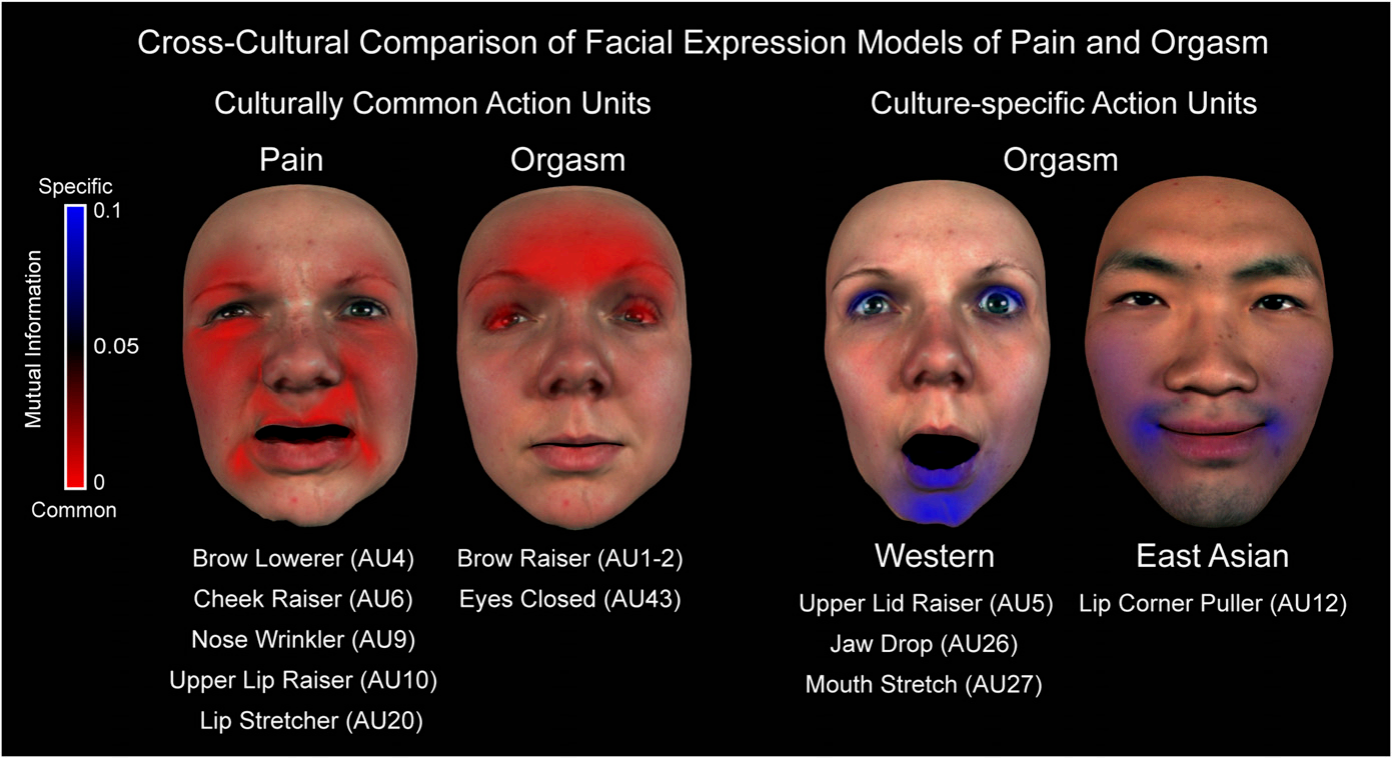
Cross-cultural comparison of facial expression models of pain and orgasm. To identify any cross-cultural and culture-specific action units, we used MI to measure the relationship between AUs and culture (*Methods*, *Cross-Cultural Comparison of the Facial Expression Models of Pain and Orgasm*). Each color-coded face map shows the AUs that are common across cultures (red, low MI) or specific to one culture (blue, high MI, *P* < 0.05; see color bar to the left). As shown by the red coloring, pain shows several cross-cultural face movements (see AU labels below) and no culture-specific face movements. Orgasm also showed cross-cultural face movements (e.g., brow raiser, AUs 1 and 2) with culture-specific accents such as jaw drop (AU26) and mouth stretch (AU27) among Westerners, and lip corner puller (AU12) among East Asians.

Using a combination of Bayesian classification, human perceptual discrimination, and an information-theoretic analysis, we showed that the facial expression models of pain and orgasm are distinct in each culture. One outstanding question is whether the different representations of pain and orgasm are the same across cultures, because identifying cultural similarities and differences in social communication is critical to understanding the diversity and fundamental basis of human interaction (36–40). To examine this, we used the same information-theoretic approach described above (i.e., MI analysis applied to highly frequent AUs) to measure the relationship between AUs and culture (see *Methods*, *Cross-Cultural Comparison of the Facial Expression Models of Pain and Orgasm* for full details). Fig. 4 shows the results. AUs that are common across cultures are indicated by red coloring (low MI values); AUs that are specific to one culture are indicated by blue coloring (high MI values; see color bar to the left). The results show that across cultures, the facial expression models of pain comprise highly similar face movements including brow lowerer (AU4), cheek raiser (AU6), nose wrinkler (AU9), upper lip raiser (AU10), and lip stretcher (AU20). In contrast, the facial expression models of orgasm comprise different face movements across cultures: Western models include upper lid raiser (AU5), jaw drop (AU26), and mouth stretch (AU27), whereas East Asian models include lip corner puller (AU12). As shown by the blue coloring, these culture-specific face movements are also combined with cross-cultural AUs including brow raiser (AUs 1 and 2) and eyes closed (AU43).

### Comparison of the Mental Representations and Productions of Facial Expressions of Pain and Orgasm.

Here, we compare our facial expression models with the face movements reported in real-world production studies. To benchmark, we compiled 11 studies on the production of facial expressions during pain (20–30) and 3 studies for orgasm (1–3). We report results only using Western data, because sufficient production data are not yet available for East Asians (but see also refs. 1 and 41). For each production study, we extracted all AUs reported as produced during pain or orgasm, thereby producing 11 pain AU patterns and 3 orgasm AU patterns (see *SI Appendix*, *Comparison of the Mental Representations and Productions of Facial Expressions of Pain and Orgasm* and Fig. S1 for methodology and full details).

#### Classification of mental representations of facial expressions by real-world productions.

**Fig. 5.**
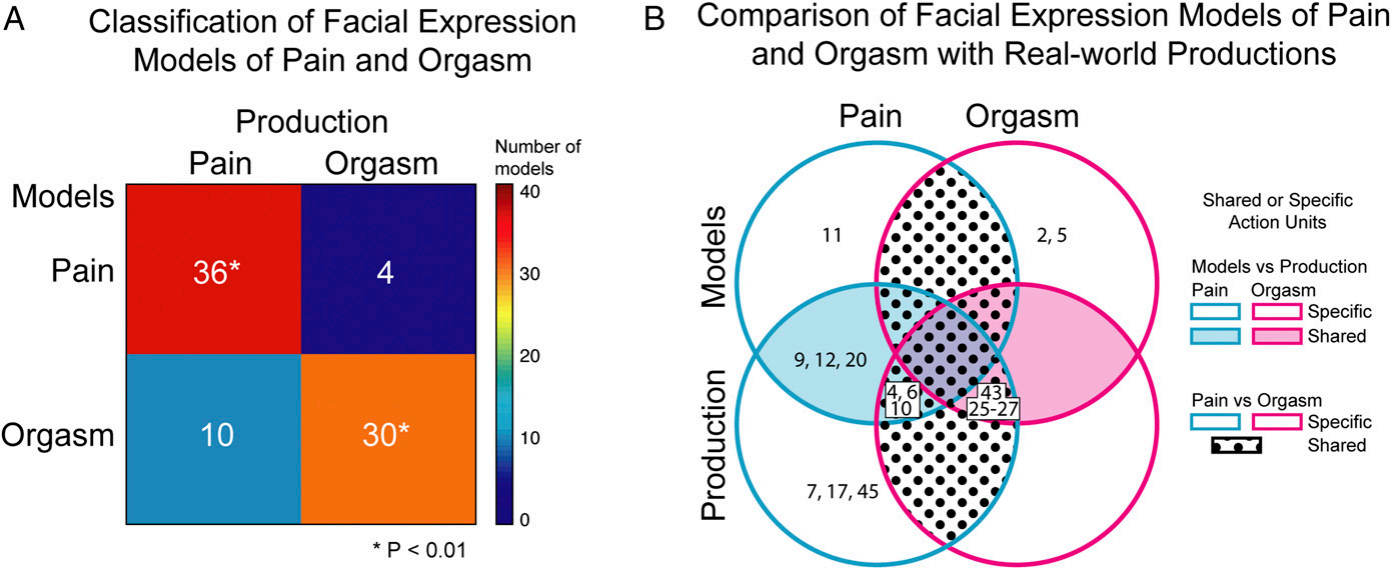
Comparison of mental representations and productions of facial expressions of pain and orgasm. (*A*) Classification of facial expression models of pain and orgasm. The color-coded matrix shows the number of facial expression models (*n* = 40 total) classified as pain or orgasm based on their highest similarity to real-world productions of facial expressions of pain and orgasm (*n* = 14 produced facial expressions in total). Diagonal squares show correct classifications; off-diagonal show incorrect classifications. Warmer colors indicate higher numbers of models; cooler colors indicate lower numbers (see color bar to the right). Exact numbers are shown in each cell. As shown by the diagonal squares, the vast majority of pain and orgasm facial expression models are correctly classified based on the face movements reported in real-world production studies. (*B*) Comparison of facial expression models of pain and orgasm with real-world productions. The four-set Venn diagram shows the AUs that converge or diverge across the four sets of AUs, reported in facial expression models and produced facial expressions of pain (cyan) and orgasm (magenta). Textured areas show the AUs that are shared between pain and orgasm; shaded areas show the AUs that are shared between models and productions of facial expressions; untextured/unshaded areas show the AUs that are specific to one of the sets (see key to the right).

First, we tested whether the facial expression models are accurately classified as pain or orgasm based on knowledge of their real-world production. Specifically, we measured the similarity between the facial expression models and their real-world productions using the Hamming distance, which measures the number of dimensional values that differ between two (here, binary) vectors (42). For all facial expression models (*n* = 80 total), we classified each as pain or orgasm based on its highest similarity (i.e., lowest Hamming distance) to the AU patterns reported in all production studies (*n* = 14 AU patterns total). Fig. 5*A* shows the results. The color-coded confusion matrix shows the number of facial expression models (*y* axis) classified as pain or orgasm (*x* axis). Diagonal squares show correct classifications; off-diagonal squares show incorrect classifications. Warmer colors indicate a higher number of facial expression models; cooler colors indicate lower numbers (see color bar to the right). Exact numbers are shown in each cell. As shown by the diagonal squares, the vast majority of both pain and orgasm facial expression models are correctly classified based on knowledge of their real-world production [Χ^2^ (1, *n* = 80) = 34.58, *P* < 0.01].

#### Similarities and differences in face movements between mental representations and produced facial expressions of pain and orgasm.

Next, we examined the similarities and differences in face movements between the mental representations and productions of facial expressions of pain and orgasm. We included all AUs reported in the majority of production studies or mental representations (*SI Appendix*, Fig. S1*A*). Fig. 5*B* presents these AUs as a four-set Venn diagram with pain (cyan) and orgasm (magenta) represented on the horizontal axis and models and production studies presented on the vertical axis. Action units shared between pain and orgasm are shown in the textured areas, whereas AUs shared between the models and productions are shown in the shaded areas. Action units that are specific to one set (e.g., produced facial expressions of pain) are shown in the untextured/unshaded areas (see key to the right). As shown by the top textured area, no AUs are shared between models of pain and orgasm, as reported earlier. In contrast, several AUs—4, 6, 10, 43, and 25 to 27—are shared between real-world productions of pain and orgasm, which confirms their reported similarity (see bottom textured area). However, a majority of production studies also report that AUs 7, 17, and 45 are displayed during pain but not orgasm (see bottom left, unshaded). Together, these data suggest that produced facial expressions of pain and orgasm have distinctive face movements but are less distinctive than mental representations.

Next, as shown by the shaded blue area, several AUs converge between the models and production of pain—that is, AUs 4, 6, 9, 10, 12, and 20—with a few AUs that are specific to the models (AU11) and productions (AUs 7, 17, 45). Although there are fewer orgasm production data (*n* = 3) to build a reliable comparison with the models, AUs 43 and 25 to 27 are convergent whereas AUs 2 and 5 are only present in the models. We return to these similarities and differences between models and productions in *Discussion*.

## Discussion

Here, we examined whether facial expressions of the extreme positive and negative affective states of physical pain and sexual pleasure form distinct representations in the minds of observers in two cultures. Using a data-driven technique, we mathematically modeled the mental representations of dynamic facial expressions of physical pain and orgasm in individuals from Western and East Asian cultures. We then used a complementary approach of machine learning, a human perceptual discrimination task, and an information-theoretic analysis to show that, in each culture, mental representations of the facial expressions of pain and orgasm are distinct. Furthermore, a cross-cultural analysis showed that mental representations of pain share similar face movements across cultures including brow lowering, cheek raising, nose wrinkling, and mouth stretching. In contrast, mental representation of orgasm comprised culture-specific face movements—Westerners included wide-open eyes and a vertically stretched mouth, whereas East Asians included smiling—which were combined with cross-cultural face movements such as brow raising and closed eyes. Together, these data show that mental representations of the extreme positive and negative affective states of physical pain and orgasm are distinct in the two cultures. We now discuss the implications of these results in relation to evidence from real-world production studies that show that people experiencing physical pain and orgasm produce similar facial expressions.

### Implications of Distinct Mental Representations of Facial Expressions of Pain and Orgasm.

Our results from modeling the mental representations of facial expressions of pain and orgasm show that they are distinct. Specifically, we show in both cultures that mental representations of pain and orgasm comprise opposing face movements—whereas pain is characterized by those that contract the face inward (e.g., brow lowering, nose wrinkling, and cheek raising), orgasm is represented by face movements that expand the face outward (e.g., brow raising in both cultures; mouth opening and eyelid raising among Westerners). Such contrasting face movements are therefore prime candidates for communicating these different affective states to others (43) and to influence their behavior—for example, eliciting help in the case of pain or indicating completion of a sexual act in orgasm. Disentangling which face movements serve social communication and which are primarily a physiological response requires further understanding of how social contexts (e.g., dyads) influence facial behaviors in different cultures. In either case, our data show that distinct facial expressions can be used to convey the extreme affective states of pain and orgasm in both cultures. Although not studied here, transient changes in facial coloration such as blushing and pallor could comprise a key component of the facial behavior produced during pain and orgasm and thus contribute to the perception of these intense affective states in others (e.g., see refs. 44 and 45). We anticipate that such questions will soon be addressed in future research.

### Mental Representations Versus Real-World Production of Facial Expressions.

We show that mental representations of pain and orgasm share many face movements with their real-world productions, suggesting that mental representations are statistically related to real-world displays. This is further supported by the models being recognized as pain and orgasm by a separate group of observers in each culture. However, our finding that mental representations of facial expressions of pain and orgasm are distinct contrasts with real-world studies of the production of these facial expressions, which report that they are similar. Specifically, productions of pain and orgasm share several face movements such as brow lowering, cheek and lip raising, eye closing, and mouth opening, and differ on others such as wincing, chin raising, and blinking. This suggests that although produced facial expressions of pain and orgasm show distinctive features, mental representations are even more distinctive than their real-world displays. This discrepancy could arise from specific divergences between mental representations and real-world displays. For example, our comparison analysis suggests that mental representations comprise a subset of the most diagnostic face movements—for example, facial expression models of pain include most AUs reported in produced facial expressions such as brow lowering, nose wrinkling, and horizontal lip stretching, but not AUs such as chin raising or wincing. Such efficient encoding of the most diagnostic and visually salient face movements could facilitate memory storage and perceptual categorization in the real world. Relatedly, mental representations could also represent supernormal stimuli where certain features of real-world displays are exaggerated, and which could draw more attention to these features in the environment as a result (46, 47).

Divergence could also arise due to the influence of other concepts such as idealized behaviors, that is, those that have a high value within a culture. For example, our results show that East Asian mental representations of facial expressions of orgasm include smiling, whereas Western models show a wide-open mouth. These cultural differences correspond to current theories of ideal affect (48) that propose that Westerners value high arousal positive states such as excitement and enthusiasm, which are often associated with wide-open eye and mouth movements (2, 3, 40), whereas East Asians tend to value low arousal positive states, which are often associated with closed-mouth smiles (49). As discussed in current theories of ideal affect, cultural ideals influence the behaviors of individuals within that culture—that is, Westerners are expected to display positive states as high arousal (e.g., excited), whereas East Asians are expected to display positive states as low arousal (e.g., calm). Therefore, it is likely that Westerners and East Asians display different facial expressions in line with the expectations of their culture. Indeed, we show that Western mental representations of orgasm share AUs with produced facial expressions (e.g., AUs 43 and 25 to 27), which by extension suggests that East Asians might produce these facial expressions during orgasm. We anticipate that such questions could be addressed when sufficient East Asian production data become available. Similarly, mental representations could also reflect the influence of social motives, values, semantic knowledge, or pragmatic competence (6, 11, 12), which could also shape real-world displays.

A further source of divergence between mental representations and real-world displays could be variance in experimental conditions. For example, the mental representations reported here comprise a dynamic facial expression displayed over a relatively short time period (2.25 s), whereas some production studies capture face movements displayed over longer periods or during a single snapshot. Such variance in recording methods could therefore capture different segments of dynamically evolving facial display or a series of different facial displays that represent different stages of experiencing pain or sexual pleasure (see *SI Appendix*, Fig. S1 for study details).

In all cases discussed above, a better understanding of the nature and origin of the divergences and/or biases in mental representations requires detailed comparisons with the “ground truth”—that is, knowledge of the true variety of facial expressions of pain and orgasm that are displayed in the real world, including variability across time and within different cultures and social contexts (50). We anticipate that such data will become more available with the increasing development of technologies that can be used to systematically record and decode face movements in the wild.

## Conclusions

We found that mental representations of facial expressions of the extreme negative and positive states of physical pain and orgasm are distinct in two different cultures. Our results therefore question the nondiagnosticity of these facial expressions and suggest that they could serve as effective tools for social communication and interaction (4, 13). Our results also address existing questions of whether culture influences how facial expressions are represented and used to communicate basic social messages (51, 52).

Finally, understanding the ontology of facial expressions—that is, the form of face movement patterns—is a substantial question due to the complexity of the social world and the multiple variables that could influence communication (53). Our data highlight the relevance of controlling potential (and known) variables when examining the form and function of signals, such as the nature of the social context and the communication channel such as viewing distance (54). We anticipate that the development of new methods that can precisely control these potential variables and measure their contribution will allow better navigation of the complex social world and provide a richer, more accurate account of social communication.

## Methods

### Observers.

To model the mental representations of facial expressions of physical pain and orgasm in each culture, we recruited a total of 80 observers (40 Westerners, white European, 20 females, mean age 22 y, *SD* = 2.68 y; 40 East Asians, Chinese, 20 females, mean age 23 y, *SD* = 1.80 y). For the perceptual discrimination task, we recruited a new group of 104 observers (52 Western, white European, 26 females, mean age 22 y, *SD* = 2.73 y; 52 East Asians, Chinese, 26 females, mean age 23 y, *SD* = 1.54 y). To control for the possibility that the observer’s mental representations or interpretation of these facial expressions could have been influenced by cross-cultural interactions, we recruited observers with minimal exposure to and engagement with other cultures (55) as assessed by questionnaire (*SI Appendix*, *Screening Questionnaire*). We also recruited observers who were sexually active (as per self-report) and identified as heterosexual as assessed by the Kinsey scale (56) (*SI Appendix*, *Kinsey Scale*). All East Asian observers had arrived in the United Kingdom for the first time with an average UK residence of 3 mo at the time of testing (*SD* = 1.9 mo) and had a minimum International English Testing System score of 6.0 (competent user). All observers had normal or corrected-to-normal vision and were free from any emotion-related atypicalities (autism spectrum disorder, depression, anxiety), learning difficulties (e.g., dyslexia), synesthesia, and disorders of face perception (e.g., prosopagnosia) as per self-report. We obtained each observer’s written informed consent before testing and paid each observer £6 per h for their participation. The University of Glasgow, College of Science and Engineering Ethics Committee authorized the experimental protocol (reference ID 300140074).

### Modeling Dynamic Mental Representations of Facial Expressions of Pain and Orgasm.

All observers completed the facial animation categorization task as illustrated in Fig. 1. We instructed observers to categorize each facial animation according to physical pain defined as “the sharp sensory pain when receiving, for example, an electroshock, keeping a limb sunken in icy water, or back pain” or orgasm defined as “the brief and intense experience during the sexual response cycle after the first arousal phase and the sustained sexual excitement phase.” We provided participants with a figure illustrating the different phases of the sexual response cycle—that is, excitement, plateau, orgasm, and resolution. To compute models of the dynamic facial expressions of pain and orgasm for each individual observer, we used an established model-fitting procedure (18). First, we performed a Pearson correlation between two binary vectors: The first vector detailed the presence or absence of each AU on each trial; the second detailed the response of the observer (pain = 0, orgasm = 1). For all significant correlations (two-tailed, *P* < 0.05), we assigned a value of 1 (0 otherwise), thus producing a 1 × 42-dimensional binary vector detailing the composition of AUs that are significantly associated with the perception of each affective state for that observer. To model the dynamic components of each significant AU, we performed a linear regression between the second binary response variable and the seven temporal parameters of each significantly correlated AU, as detailed on each trial. To calculate the intensity gradients of each of the facial expression models, we fitted a linear regression model to the temporal parameters of each significantly correlated AU and the observer’s intensity ratings. To make the resulting dynamic face movements into movies for later use as stimuli, we then combined the significantly correlated AUs with their temporal activation parameters, using only the “high-intensity” ratings, as these comprise the most salient signals (see Movie S2 for an illustration of the procedure). Our approach therefore delivers the precise dynamic facial expressions that elicit the perception of pain and orgasm in each individual observer in each culture.

### Physical and Perceptual Distinctiveness of the Facial Expression Models of Pain and Orgasm.

For each culture and sex of observer (total 104 observers; 2 cultures × 2 sexes × 26 observers), we displayed a set of 40 same-culture, same-sex facial expression models of pain and orgasm (20 models × 2 pain/orgasm) on 10 new same-race, sex-opposite face identities (20 white European, 10 females, mean age 22 y, *SD* = 3.49 y; 19 Chinese, 1 Japanese, mean age 24 y, *SD* = 2.14 y). For example, for the new group of Western male observers, we displayed the 40 facial expression models of pain and orgasm derived from Western male observers in *Modeling Dynamic Mental Representations of Facial Expressions of Pain and Orgasm* on 10 white female faces. Thus, for each culture and sex of new observers, we generated 400 facial expression stimuli (20 same-culture, same-sex facial expression models × 2 pain/orgasm × 10 same-race, sex-opposite face identities).

On each experimental trial, observers first viewed a word (pain or orgasm) displayed on-screen for 1.5 s and followed directly by either a correctly or incorrectly matched facial expression displayed once for 2.25 s. We asked observers to indicate whether or not the preceding word accurately described the facial expression by pressing yes or no keys on a keyboard and to respond as accurately as possible. We assigned yes and no keys to separate hands for each observer and counterbalanced key assignments across observers. Half of the trials comprised correct word–facial expression matches and included all 400 facial expression stimuli, with the other half of the trials comprising incorrect word–facial expression matches. Each observer therefore completed 800 trials (400 facial expression stimuli × correct/incorrect matches) presented in random order across the experiment. We used the same stimulus presentation display as used in *Modeling Dynamic Mental Representations of Facial Expressions of Pain and Orgasm*. Each observer completed the experiment over four ∼20-min sessions with a short break (∼5 min) in-between sessions. On average, observers completed the experiment in 1.25 h (*SD* = 0.25 h) in 1 d. Following the experiment, we computed the *d*-prime of each individual facial expression model in each culture by pooling the responses from all observers who completed the perceptual discrimination task.

### Comparison of the Facial Expression Models of Pain and Orgasm.

To identify the AUs that are specific to or common across the facial expression models of pain and orgasm in each culture, we computed the mutual information for each highly frequent AU and established statistical significance using a Monte Carlo approach. Specifically, for each highly frequent AU, we produced a random distribution of MI values by randomly shuffling the affective state assignment (i.e., pain or orgasm) of each individual facial expression model 1,000 times, computing the MI for each AU at each iteration, and then taking the maximum MI value across all AUs. We then used the distribution of maximum MI values to identify the AUs with an MI value in the 95th percentile of the distribution (57).

### Cross-Cultural Comparison of the Facial Expression Models of Pain and Orgasm.

To examine whether the facial expression models of pain and orgasm are similar or different across cultures, we applied MI analysis between the AUs and culture for pain and orgasm separately. To establish statistical significance, we used a Monte Carlo approach as above but by randomly shuffling the cultural assignment (i.e., Western or East Asian) of the facial expression models.

## Supplementary Material

Supplementary File

Supplementary File

Supplementary File

Supplementary File
