# Supplementary material for: Distinct facial expressions represent pain and pleasure across cultures
Source: Proc Natl Acad Sci U S A. 2018 Oct 8;115(43):E10013–21. doi: 10.1073/pnas.1807862115 (PMC6205428; doi:10.1073/pnas.1807862115)
Supplement: Supplementary File [file pnas.1807862115.sapp.pdf]

PNAS
www.pnas.org

Supplementary Information for

Distinct Facial Expressions Represent Pain and Pleasure Across Cultures

Chaona Chen, Carlos Crivelli, Oliver G. B. Garrod, Philippe G. Schyns, José-Miguel Fernández-Dols, Rachael E. Jack

Rachael E. Jack
Email: rachael.jack@glasgow.ac.uk

This PDF file includes:

Supplementary text
Figs. S1 to S2
Captions for movies S1 to S3
References for SI reference citations

Other supplementary materials for this manuscript include the following:

Movies S1 to S3

Supplementary Information Text
Screening questionnaire
Each potential participant completed one of the following questionnaires depending on their cultural background. We selected only those who answered 'no' to all questions for participation in the experiment.

Western observers

1. Have you ever lived in non-Western* country before (e.g. on a gap year, summer work, move due parental employment)?
2. How many weeks have you spent in a non-Western country (e.g. on vacation)?
3. Have you ever dated or had a very close friendship with a non-Western person?
4. Have you ever been involved with any non-Western culture societies/groups?

*by Western groups/countries, we are referring to Europe (East and West), USA, Canada, United Kingdom, Australia and New Zealand.

East Asian observers

1. Have you ever lived in non-East Asian* country before (e.g. on a gap year, summer work, move due parental employment)?
2. How many weeks have you spent in a non-East Asian* country (e.g. on vacation)?
3. Have you ever dated or had a very close friendship with a non-East Asian* person?
4. Have you ever been involved with any non-East Asian* culture societies/groups?

*by East Asian groups/countries, we are referring to China, Japan, Korea, Thailand and Taiwan.

Kinsey scale
Each potential participant completed the following questionnaire about his or her sexual orientation. The Kinsey Scale ranges from 0 (exclusively heterosexual) to 6 (exclusively homosexual). We selected only those who answered 'exclusively heterosexual' for participation in the experiment.

Please indicate your sexuality using one the decriptions below

- Exclusively heterosexual
- Predominantly heterosexual, only incidentally homosexual
- Predominantly heterosexual, but more than incidentally homosexual
- Equally heterosexual and homosexual
- Predominantly homosexual, but more than incidentally heterosexual
- Predominantly homosexual, only incidentally heterosexual
- Exclusively homosexual
- Non-sexual

Modelling dynamic mental representations of facial expressions of pain and orgasm

For each culture and sex of observer (20 males and 20 females in each culture), we generated 3,600 random facial animations as illustrated in Fig. 1 and displayed each on one of 4 same-race, sex-opposite face identities (8 European, 4 female, mean age = 28 years, *SD* = 3.85 years; 8 Chinese, 4 female, mean age = 27 years, *SD* = 3.16 years) captured using a high-resolution 3D face capture system, see (1) for full details. We generated all stimuli using a standard procedure (1) in 3D Studio Max. All observers completed all 3,600 trials as illustrated in Fig. 1. We presented all stimuli in random order across the experiment for each observer. All stimuli (mean size = 19.12 cm [*SD* = 0.90 cm] × 13.07 cm [*SD* = 0.86 cm]) appeared in the observer's central visual field and played only once for a duration of 2.25 seconds. A chin rest ensured a constant viewing distance of 77 cm, with stimuli subtending 14.16° (vertical) × 9.70° (horizontal) of visual angle, which reflects the average size of a human face (2) during typical social interaction (3). We presented all labels (mean size = 0.96 cm [*SD* = 0.05 cm]) × 2.63 cm [*SD* = 0.44 cm]) in lower case white font ('MS Sans Serif') and in English, thus subtending 0.72° × (vertical) × 1.96° (horizontal) of visual angle, with all stimuli presented on a black background displayed on a 19-inch flat panel Dell monitor with a refresh rate of 60 Hz and resolution of 1024 × 1280. Each observer completed the experiment over eighteen ~20-minute sessions with a short break (~5 minutes) after each session. After one hour (e.g., 3 concurrent sessions), observers took a longer break of at least 1 hour. Observers completed the entire experiment over 2 or 3 days (mean = 6 sessions per day, *SD* = 3 sessions). On average, observers completed the whole experiment in 6.75 hours (*SD* = 0.75 hours).

Highly frequent action units

For pain and orgasm and for each culture separately, we identified the AUs that are highly frequent across the facial expression models using a Monte Carlo simulation method. To illustrate, consider we aim to identify the AUs that are highly frequent across the Western facial expression models of pain. First, in the total set of 40 Western models × 42 AUs, we computed the frequency of each AU across all 40 models. For example, brow lowerer (AU4) is present a total of 31 times over all models, resulting in a 31/40 frequency. We also computed the total number of significant AUs across all individual observer models. For example, there are a total of 232 significant AUs across the 40 Western facial expression models of pain. Next, to determine the statistical significance of each AU frequency, we used a Monte Carlo simulation method to randomly distribute (with replacement) the total number of significant AUs (here, 232 AUs) over the space of 40 Western models × 42 AUs and then compute the resulting frequency of each AU. For example, on this iteration, inner brow raiser (AU1) might have a frequency of 7/40. Over 1,000 iterations, we thus derive a distribution of AU frequencies, which we used to test the null hypothesis that the observed 31/40 proportion of brow lowerer (AU4) in the Western facial expression models of pain is significantly higher than chance—i.e. above the 95th percentile of the randomly generated distribution of AU frequencies (i.e. one-tailed $p < .05$). If so, we call brow lowerer (AU4) a *highly frequent* AU. We repeated this procedure for each AU and for pain and orgasm and for each culture separately.

Comparison of the mental representations and productions of facial expressions of pain and orgasm

To examine whether mental representations of facial expressions of pain and orgasm are similar to those produced in the real-world, we compared the individual facial expression models of pain and orgasm to the facial expressions of pain and orgasm reported in production studies. All pain production data are extracted from 11 studies (4-14) and all orgasm production data are extracted from 3 studies (15-17). We did not include studies that used AU patterns reported in previous studies (e.g., 18, 19). We report results only using Western data because sufficient production data are not yet available for East Asians (but see also 16, 20). Before presenting our results, two caveats are necessary. First, although a reasonable number of pain production studies (n = 11) exist to estimate real-world variance and thus to compare with mental representations, few production studies on orgasm currently exist (n = 3) to draw robust conclusions. Second, production studies vary in the experimental conditions and protocols used – for example, some recorded AUs from video clips (i.e., several frames) (4-9, 11-13, 15, 17) whereas others use images (i.e., a single frame) (10, 16); pain induction methods also differ – for example, cold compress (12-14), electric shock (4, 12), examination of an injury (5-8, 11) – that could elicit different types of pain. This variation therefore makes comparing results across these different studies and with the current study problematic because variation in the reported AU patterns could arise from differences in the experimental conditions. Consequently, we present our analysis comparing the mental representations of facial expressions of pain and orgasm with production studies with these cautionary notes.

Action Units reported in production studies of facial expressions of pain and orgasm. For each production study, we extracted all AUs reported as produced during pain or orgasm and represented each as a binary vector (1 = AU produced; 0 = AU not produced), thereby producing 11 pain AU patterns and 3 orgasm AU patterns. Fig. S1 Panel A shows each set of AU patterns for pain and orgasm separately as a black and white matrix. Above each, a number represents the specific (see key at bottom) with details of the study provided below. To the right of each matrix, the first color-coded vector shows the proportion of studies with each AU, with warmer colors indicating higher numbers and cooler colors indicating lower numbers (see colorbar to the right). Black dots represent AUs that are present in the majority of studies. A visual inspection of the AU patterns shows both consistency and variation across studies – for example, in pain, cheek raiser (AU6) is reported in 11/11 studies whereas inner brow raiser (AU1) is reported in 6/11 studies – suggesting that facial expressions of pain and orgasm could comprise different sub-types. The second color-coded vector shows the proportion of models with each AU with black dots representing AUs that are present in the majority of models. This also shows that some AUs are more prevalent across the models than others – for example, in pain, lip stretcher (AU20) is shown in 38/40 models where lid tightener (AU7) is shown in 13/40 models.

Comparison of mental representations of facial expressions with action units reported in production studies. To examine whether the facial expression models correspond with real-world productions, we measured for pain and orgasm separately, the similarity (1 – Hamming distance) between each facial expression model (n = 40) and the AU pattern reported in each production study (n = 11 pain, n = orgasm). Panel B shows the results. Face maps represent the AUs reported in each production study ranked by their average (median) similarity to the 40 facial expression models from

most similar (left) to least similar (right). Median similarity is shown below each face. Below, we also show for each production study, the number of facial expression models that are most similar (lowest Hamming distance) to the AU patterns reported in that study – for example, over half (n = 22) of the models are most similar to the AUs reported in study 1 with 11 models most similar to study 3. Together, these results suggest that the facial expression models are most similar to a sub-set of produced facial expressions. Next, to highlight differences between the models and the AUs reported in each production study, we list the AUs that appear exclusively in the produced facial expression (top row) or in the facial expression models (bottom row) for each study separately. For the model-specific AUs, we list the AU that occurs most frequently across the models. For example, in study 1, blink (AU45) is present in the produced facial expression but not in any of the facial expression models, whereas lip stretcher (AU20) is not present in the produced facial expression but appears in 38/40 facial expression models. Note that all model-specific AUs are reported in at least one other production study. For, example, in study 1, lip stretcher (AU20) is not reported in study 1 but appears in 8/11 other production studies (5-8, 10-13). For the production-specific AUs, all but two AUs – inner brow raiser (AU1) and outer brow raiser (AU2) – were not included in the stimulus generation procedure – i.e., blink (AU45), lid droop (AU41), lip pucker (AU18), tongue out (AU19).

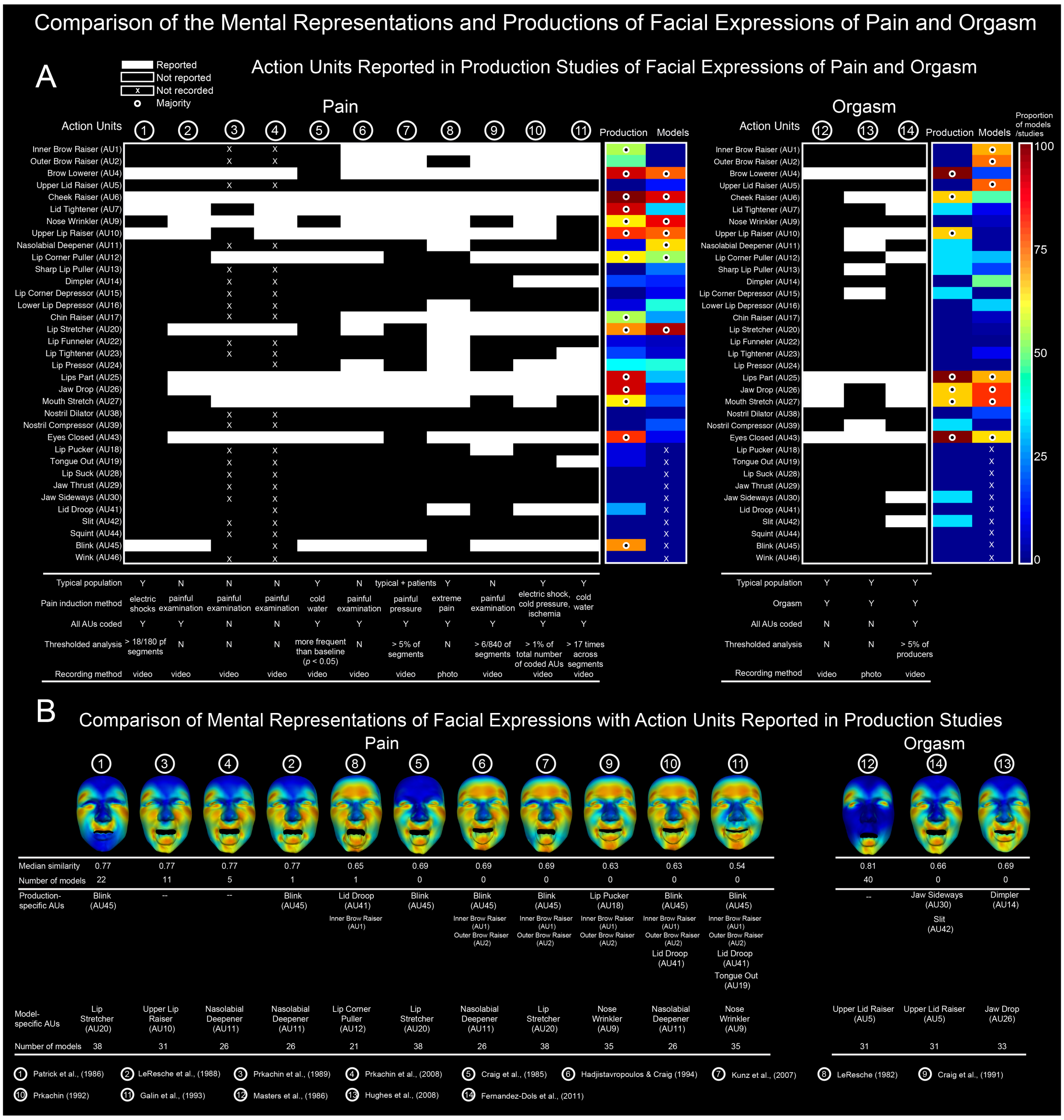

Fig. S1. Comparison of the mental representations and productions of facial expressions of pain and orgasm. *(A) Action units reported in production studies of facial expressions of pain and orgasm.* For pain and orgasm separately, the black and white matrices show the AUs reported in each production study. White indicates the AU was reported, black that it was not reported, ‘x’ indicates the AU was not recorded (see legend in top left; AU labels on the left). Above each, a number represented the specific study (see key at bottom). Details of each study – for example, the method of pain induction – are provided below. To the right of each matrix, the first color-coded vector shows the proportion of studies with each AU; the second reports the proportion of models with each AU. Warmer colors indicate a high proportion, cooler colors indicate a low proportion (see colorbar to right). Black dots indicate the AUs reported in a majority of studies/models. *(B) Comparison of mental representations with action units reported in production studies.* Face maps represent the AUs reported in each study, ranked according to their median similarity (1 – Hamming distance) to the facial expression models (most similar – left; least similar – right). The median similarity and number of models most similar (lowest Hamming distance) to the AUs in each study are shown below. AUs appearing exclusively in produced facial expressions or in the models are listed below each face (for model-specific AUs, the most frequent AU across the models is reported).

Bayesian classification of facial expression models

To objectively determine the distinctiveness of the facial expression models of pain and orgasm in each culture, we built a Bayesian model of the discrimination task using a split-half method. Specifically, for each culture separately, we trained a Bayesian classifier on half of the facial expression models of pain and half of orgasm selected randomly (each model is represented as a 1×42 binary vector detailing the composition of significant AUs). We then tested the classification performance of the Bayesian model on the remaining half of the facial expression models in a pain vs. orgasm discrimination task. We repeated this procedure 1,000 times and computed the average Bayesian posterior probability of pain or orgasm classifications. Fig. 3*A* shows the results.

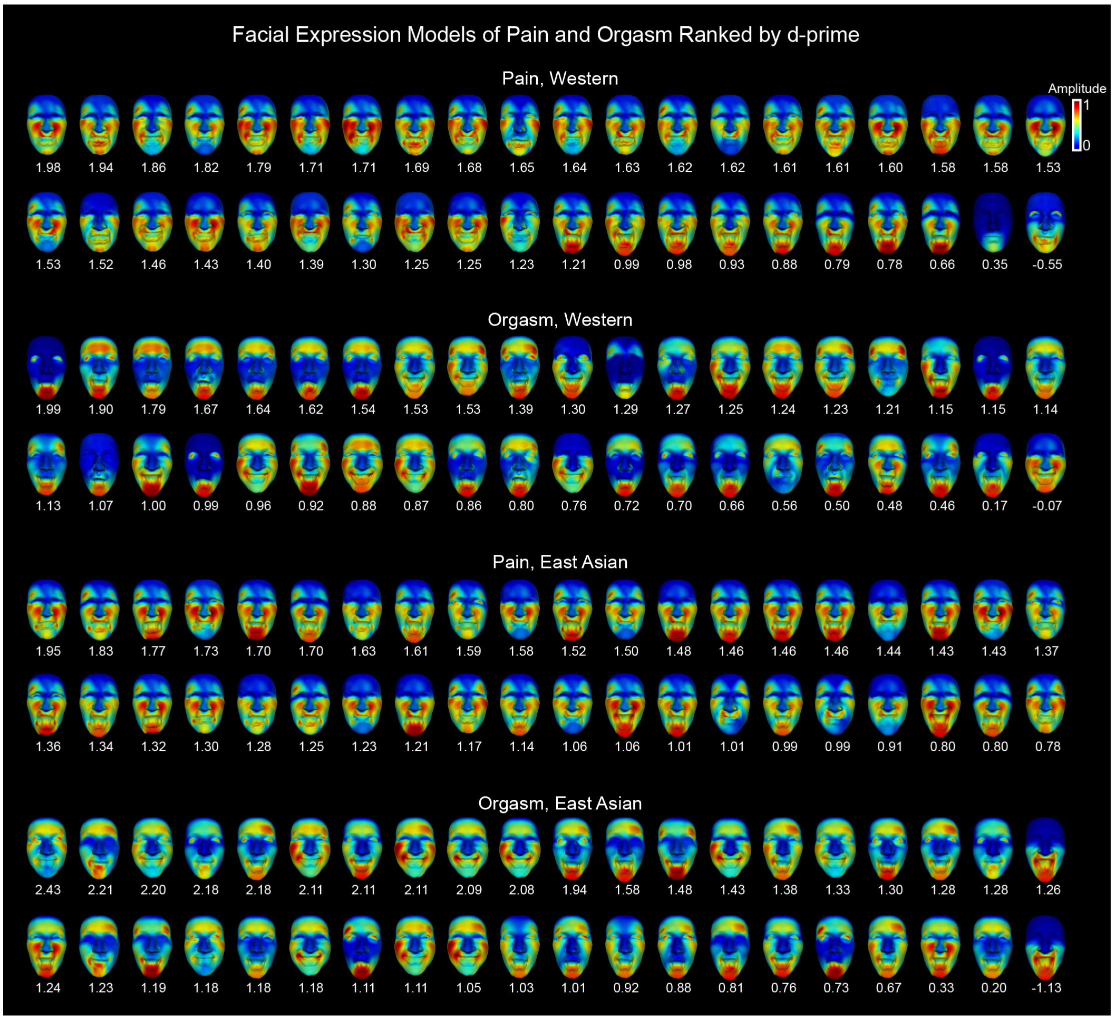

Fig. S2. Facial expression models of pain and orgasm from each culture ranked by d-prime. Color-coded face maps show the individual facial expression models of pain and orgasm for each culture. Color-coding represents the amplitude (i.e., intensity) of each significant Action Unit (see colorbar in top right) as derived from the linear regression analysis. Facial expression models are ranked according to d-prime (see values below each face map) in descending order from left to right.

Movie S1. The movie illustrates the stimulus generation and task procedure using an example trial. On each trial, a dynamic face movement generator (1) randomly selected a combination of individual face movements called action units (AUs; 21) from a core set of 42 AUs (minimum = 1, maximum = 4, median = 3 AUs selected on each trial). A random movement is assigned to each AU individually using seven randomly selected temporal parameter values – onset latency, acceleration, peak amplitude, peak latency, sustainment, deceleration, and offset latency. In this example trial, four AUs are randomly selected – brow lower (AU4) color-coded in yellow, cheek raiser (AU6) color-coded in blue, nose wrinkler (AU9) color-coded in pink, and lip stretcher (AU20) color-coded in red. The randomly activated AUs are then combined to produce a random facial animation (here, 'Stimulus trial'). Observers in each culture viewed the resulting facial animation played once for a duration of 2.25 seconds. If the random face movements matched their mental representation of a facial expression of 'pain' or 'orgasm,' they categorized it accordingly (here, 'Pain') and rated its intensity on a 5-point scale from 'very weak' to 'very strong' (here, 'Strong').

Movie S2. The movie illustrates the procedure to model dynamic mental representations of facial expressions of 'pain' and 'orgasm.' For each individual observer, we obtained the trials they categorized as 'pain' or 'orgasm' and computed the AUs significantly associated with the perception of each affective state using correlation. We then used linear regression to derive the temporal parameters of each significant AU. To represent the resulting dynamic mental representations of pain and orgasm, we combined the significantly correlated AUs with their temporal activation parameters. Two such examples are shown here.

Movie S3. The movie shows example dynamic mental representations of the facial expressions of 'pain' or 'orgasm' from one observer in each culture.

SI References

1. Yu H, Garrod OGB, Schyns PG (2012) Perception-driven facial expression synthesis. *Comput Graph* 36(3):152-162.
2. Ibrahimagić-Šeper L, Čelebić A, Petričević N, Selimović E (2006) Anthropometric differences between males and females in face dimensions and dimensions of central maxillary incisors. *Med Glas* 3:58-62.
3. Hall ET (1966) *The Hidden Dimension* (New York: Doubleday & Co.).
4. Patrick CJ, Craig KD, Prkachin KM (1986) Observer judgments of acute pain: Facial action determinants. *J Pers Soc Psychol* 50(6):1291.
5. LeResche L, Dworkin SF (1988) Facial expressions of pain and emotions in chronic TMD patients. *Pain* 35(1):71-78.
6. Prkachin KM, Mercer SR (1989) Pain expression in patients with shoulder pathology: validity, properties and relationship to sickness impact. *Pain* 39(3):257-265.
7. Prkachin KM, Solomon PE (2008) The structure, reliability and validity of pain expression: Evidence from patients with shoulder pain. *Pain* 139(2):267-274.
8. Hadjistavropoulos HD, Craig KD (1994) Acute and chronic low back pain: Cognitive, affective, and behavioral dimensions. *J Consult Clin Psychol* 62(2):341-349.
9. Kunz M, Scharmann S, Hemmeter U, Schepelmann K, Lautenbacher S (2007) The facial expression of pain in patients with dementia. *Pain* 133(1-3):221-228.
10. LeResche L (1982) Facial expression in pain: a study of candid photographs. *J Nonverbal Behav* 7(1):46-56.
11. Craig KD, Hyde SA, Patrick CJ (1991) Genuine, suppressed and faked facial behavior during exacerbation of chronic low back pain. *Pain* 46(2):161-171.
12. Prkachin KM (1992) The consistency of facial expressions of pain: a comparison across modalities. *Pain* 51(3):297-306.
13. Galin KE, Thorn BE (1993) Unmasking pain: detection of deception in facial expressions. *J Soc Clin Psychol* 12(2):182-197.
14. Craig KD, Patrick CJ (1985) Facial expression during induced pain. *J Pers Soc Psychol* 48(4):1080-1091.
15. Fernández-Dols J-M, Carrera P, Crivelli C (2011) Facial behavior while experiencing sexual excitement. *J Nonverbal Behav* 35(1):63-71.
16. Hughes SM, Nicholson SE (2008) Sex differences in the assessment of pain versus sexual pleasure facial expressions. *J Soc Evol Cult Psychol* 2(4):289-298.
17. Masters WH, Johnson VE (1966) *Human sexual response* (Little, Brown, and Co, Boston, MA).
18. Deyo KS, Prkachin KM, Mercer SR (2004) Development of sensitivity to facial expression of pain. *Pain* 107(1-2):16-21.
19. Hadjistavropoulos HD, Craig KD, Hadjistavropoulos T, Poole GD (1996) Subjective judgments of deception in pain expression: Accuracy and errors. *Pain* 65(2-3):251-258.
20. Rosmus C, Johnston CC, Chan-Yip A, Yang F (2000) Pain response in Chinese and non-Chinese Canadian infants: is there a difference? *Soc Sci Med* 51(2):175-184.

21. Ekman P, Friesen WV (1978) *Manual for the Facial Action Coding System* (Consulting Psychologists Press, Palo Alto, CA).
